# Novel *Siphoviridae* Bacteriophages Infecting *Bacteroides uniformis* Contain Diversity Generating Retroelement

**DOI:** 10.3390/microorganisms9050892

**Published:** 2021-04-21

**Authors:** Stina Hedžet, Maja Rupnik, Tomaž Accetto

**Affiliations:** 1 Centre for Medical Microbiology, Department for Microbiological Research, National Laboratory for Health, Environment and Food (NLZOH), 2000 Maribor, Slovenia; stina.hedzet@nlzoh.si (S.H.); maja.rupnik@nlzoh.si (M.R.); 2 Faculty of Medicine, University of Maribor, 2000 Maribor, Slovenia; 3 Animal Science Department, Biotechnical Faculty, University of Ljubljana, 1000 Ljubljana, Slovenia

**Keywords:** gut, *Bacteroides*, virome, prophage, diversity-generating retroelement

## Abstract

Intestinal phages are abundant and important components of gut microbiota, yet the isolated and characterized representatives that infect abundant gut bacteria are sparse. Here we describe the isolation of human intestinal phages infecting *Bacteroides* *uniformis*. *Bacteroides* is one of the most common bacterial groups in the global human gut microbiota; however, to date not many *Bacteroides* specific phages are known. Phages isolated in this study belong to a novel viral genus, Bacuni, within the *Siphoviridae* family. Their genomes encode diversity-generating retroelements (DGR), which were shown in other bacteriophages to promote phage adaptation to rapidly changing environmental conditions and to broaden their host range. Three isolated phages showed 99.83% genome identity but one of them infected a distinct *B. uniformis* strain. The tropism of Bacuni phages appeared to be dependent on the interplay of DGR mediated sequence variations of gene encoding putative phage fimbrial tip proteins and mutations in host genes coding for outer-membrane proteins. We found prophages with up to 85% amino acid similarity over two-thirds of the Bacuni phage genome in the *B. acidifaciens* and *Prevotella* sp. genomes. Despite the abundance of *Bacteroides* within the human microbiome, we found Bacuni phages only in a limited subset of published gut metagenomes.

## 1. Introduction

Intestinal viruses and their impact on human health are a neglected component of the widely studied gut microbiota. Bacteriophages exhibit various life styles and play an important role in shaping bacterial diversity and composition of the intestinal microbiota through predation and horizontal gene transfer [1,2]. Sequencing-based metagenomic studies have enabled insight into this complex viral reservoir, revealing genetically very diverse phages [1,3,4,5,6].

Virome metagenomic studies encounter several difficulties. The vast majority (75–99%) of sequencing reads do not correspond to any matches in existing viral databases [3]. Viruses lack universal marker genes, while standardized protocols for sample preparations and analysis are not yet established [1]. To decipher the gut virome and to connect biological characteristics with metagenomic data, cultivation of intestinal phages and their associated hosts remains crucial. A great number of intestinal phages infect anaerobic bacteria, which are challenging to cultivate; isolated and characterized phages are therefore sparse.

Despite these difficulties, several phages and prophages were lately described in different anaerobic gut microbiota representatives. An in silico-discovered viral clade, crAss-like phages, is presumably present in 50% of Western individuals and can represent up to 90% of viral metagenomics reads per individual sample [7,8]. Prediction of the suspected *Bacteroides* sp. host was confirmed by isolation of a crAss-like phage, crAss001, that infects *Bacteroides intestinalis* and exhibits a podovirus-like morphology [9]. Its life style has yet to be elucidated. CrAss-like phages are a group of genetically highly diverse phages and other *Bacteroides* species or even other bacteria may be their hosts [10].

Lysogenic (temperate) phages have been identified in genomes of *Fecalibacterium prausnitzii* [11] and *Bacteroides dorei* [12]. Extremely large gut phage genomes (540 kb), named Lak phages, that presumably infect *Prevotella* sp. were also recovered from gut metagenomes [13]. Recently, a study of temperate phage–bacteria interactions in mice gut showed that *Roseburia intestinalis* prophages influence temporal variations in the composition of gut microbiota [14]. Additionally to *Bacteroides dorei* Hankyphage [12] and crAss001 [9], four phages infecting different species within *Bacteroides* genus were isolated and sequenced. Phages B40-8 [15] and B124-14 [16] infect *Bacteroides fragilis,* while phages ϕBrb01 and ϕBrb02, originating from sewage, infect *Bacteroides* sp. bacterial hosts isolated from rumen fluid [17]. However, compared to more than 150 phages infecting *Escherichia coli* isolated from various biomes and clinical settings [18], the number of reported bacteriophages infecting *Bacteroides* is low.

Understanding the intestinal virome depends on the number of isolated, sequenced, and characterized bacteriophages and their associated hosts. The aim of the present study was to obtain and characterize the phages targeting abundant gut bacteria from *Bacteroides* genus, and to contribute to the insight into the “viral dark matter” [19] of the interactions of bacteria and viruses in human gut. Additionally, the study provides bioinformatic evidence that the host range of isolated phages may be mediated by a diversity-generating retroelement (DGR).

## 2. Materials and Methods

### 2.1. Isolation of Bacterial Strains from Human Fecal Sample

Fecal sample, obtained from a healthy volunteer, was aliquoted and further processed and stored at −80 °C. The complete isolation of bacterial strains and preparation of fecal suspension was carried out in an anaerobic workstation at 37 °C (Don Whitley Scientific, Bingley, UK).

Dilutions of homogenized fecal suspension (20% *w/v*) prepared from fresh feces and pre-reduced anaerobic YBHI broth (Brain-heart infusion media, supplemented with 0.5% yeast extract (BioLife, Milano, Italy) and 20% of rumen fluid) were plated on YBHI agar. After 72 h of anaerobic incubation at 37 °C single colonies were randomly chosen and isolated on YBHI plates to obtain pure bacterial cultures. Isolates were identified by mass spectrometry (MALDI-TOF Biotyper System, Bruker Daltonik, Bremen, Germany). Identification of *Bacteroides* strains was confirmed by 16S rRNA gene sequencing amplified with primers 27feb to 1495revb [20] and analyzed with RDP Classifier [21].

12 isolated *Bacteroides* strains (Table S1) were then used in phage screening experiments and host range experiments.

### 2.2. Phage Enrichment from Sterile Filtrate of Homogenized Fecal Sample

The fecal sample used for the phage isolation was not identical to the sample used for bacterial strain isolation, but was retrieved from the same healthy volunteer. Fresh fecal material (5 g) was resuspended in 50 mL of SM buffer with vigorous vortexing for 20 min. SM buffer contained 100 mM NaCl, 8 mM MgSO_4_, 50 mM Tris-Cl ((1 M, pH 7.5) and 0.01% (*w/v*) gelatine (2%, *w/v*)). After cooling down on ice, fecal suspension was centrifuged twice at 5400× *g* (4 °C). Supernatant was filtered twice through 0.2 µm pore cellulose acetate (CA) syringe membrane filters (Filtropur, Sarstedt, Nümbrecht, Germany). Sterile filtrate of homogenized fecal sample (fecal water) was stored at 4 °C until further use.

Phages were initially enriched in *Bacteroides* cultures. Ten different *Bacteroides* strains (Table S1) in stationary phase (1 mL) were subcultured into 9 mL of liquid sABB (Anaerobe Basal Broth, Thermo Fisher Scientific, Waltham, MA, USA, supplemented with MgSO_4_ (0.12 mM) and CaCl_2_ (1 mM)). For phage enrichment, 1 mL of fecal water was added to the inoculated media and incubated in anaerobic conditions for 24 h at 37 °C. Subsequently, 3 mL of culture media were removed and centrifuged at 5400× *g* (4 °C). Supernatant was syringe-filtered (0.2 µm pore CA, Sarstedt, Nümbrecht, Germany) and added to 9 mL of fresh sABB media, inoculated with *Bacteroides* strain in stationary phase as described before. The procedure was again repeated after 24 h. The final sterile supernatant was refrigerated (4 °C) until further used in the double-agar-layer method. Maximal storage duration was 72 h.

### 2.3. Phage Isolation from Enrichment Co-Cultures with Bacteroides

Spot assay on a double-agar-layer (DAL) was used for phage isolation from enrichment cultures. *Bacteroides* strains cultivated in liquid sABB were sampled at two different time points with optical densities OD_620_ 0.2 (T1) and OD_620_ 0.5 (T2) for further use in DAL assay. For each time point, 10-fold dilutions were made and 200 µL of each dilution was mixed with 3 mL of soft agar that was kept anaerobically at 47 °C (sABB) and poured on the pre-reduced sABB agar basal plates. After solidification, 10-fold dilutions of supernatant filtrates of phage enrichment cultures (10 µL) were spotted on solidified agar. After 24 h of incubation, plates were checked for potential lysis zones. The top agar with clear zones was harvested anaerobically with an inoculation loop and stored in 100 µL of SM puffer for 18–24 h at 4 °C, followed by centrifugation (13,000× *g*, 5 min). Supernatant was then used for further steps in phage purification and characterization.

Phages were purified from the stored spot assay supernatants by three consecutive single plaque isolation cycles using the corresponding bacterial host strain. *Bacteroides* culture (200 µL) in log growth phase was mixed with 10-fold dilutions of lysis zone supernatant and 2.5 mL of sABB soft agar and poured onto sABB agar basal plates, allowed to solidify, and incubated at 37 °C. After 18–24 h incubation, a single plaque was picked with pipette tip, transferred to SM buffer (100 µL) and left overnight at 4 °C. After 18–24 h, phage lysates were centrifuged (13,000× *g*, 5 min) and used in a plaque assay.

### 2.4. Preparation of Phage Stock Suspensions, EM Characterization and Host Range

Each isolated phage in SM buffer (100 µL) and 200 µL of respective host bacterial culture (10^7^ cfu/mL) were mixed into 3 mL soft agar, poured on solid agar plate, and incubated in anaerobic conditions up to 24 h at 37 °C. Subsequently, SM buffer (4 mL) was gently poured on confluently lysed top agar. Plates were further incubated at 37 °C for 4 h with gentle shaking. Top agar and the remains of SM buffer were scraped and centrifuged at 5400× *g* (4 °C). The supernatant was filtered through 0.2 µm pore CA syringe membrane filters (Filtropur, Sarstedt, Nümbrecht, Germany). Prepared phage suspension was transferred to U-formed centrifuge tubes suitable for ultra-centrifugation (25,000× *g*, 120 min, 4 °C) (Beckman Coulter, Optima^™^ MAX-XP, Brea, CA, USA). Pellets were resuspended in 200 µL of SM buffer and phage stock suspensions were stored at 4 °C and −80 °C.

Transmission electron microscopy was performed at the National Institute of Biology, Ljubljana, Slovenia. The phages were photographed using transmission electron microscopy with a negative staining method. The phage suspensions were deposited on formvar-coated and carbon-stabilized copper grids and stained with a 1% (*w/v*) aqueous solution of uranyl acetate. The grids were observed using transmission electron microscope CM 100 (Philips, Amsterdam, The Netherlands) operated at 80 kV and equipped with a CCD camera Orius SC 200.

Host range of isolated phages was tested with the double-agar-layer assays using 12 *Bacteroides* strains belonging to four species (Table S1).

### 2.5. Lysogen Formation Assay

Each isolated phage was cultivated with its respective host strain. Plates with formed plaques in plaque assay were incubated in anaerobic chamber at 37 °C for an additional 72 h to allow the growth of potential lysogenic strains. From the plaques formed on double-layer agar, bacterial cultures were isolated with a sterile needle or small pipette tip and inoculated on sABB agar plates to obtain pure cultures. At least 12 strains were isolated per tested bacteriophage. Sensitivity of obtained strains for isolated phages was tested with DAL spot assay described above (Figure S1).

### 2.6. Phage and Bacterial Genome Sequencing

Phage lysate (200 µL) with app. 10^9^ pfu/mL was treated with DNase I (Sigma Aldrich, St. Louis, MO, USA) at the final concentration of 0.02 mg/mL and 0.05 mg/mL RNAse A (Sigma Aldrich, St. Louis, MO, USA) and incubated for 2 h at 37 °C, followed by 10 min heat inactivation at 90 °C. Potential presence of host genomic residues was assayed with PCR using primers targeting 16 S rRNA gene [20]. Phage DNA was extracted with RTP^®^ DNA/RNA Virus Mini Kit following manufacturer’s instructions (INVITEK Molecular, Berlin, Germany). *Bacteroides* DNA was extracted using QIAamp DNA Mini Kit (Qiagen, Düsseldorf, Germany). For phage and bacterial genomes, paired-end libraries were generated using the Nextera XT Library preparation kit (IIlumina, San Diego, CA, USA) and sequenced on MiSeq (Ilumina) with 600-cycle MiSeq ReagentKit v3.

The quality of the raw sequencing reads was examined by FastQC tool Version 0.11.9 [22]. Quality trimming was done by Trimmomatic Version 0.39 [23] and overlapping paired-end reads were merged by using FLASH software, version 1.2.11 (CBB) [24]. Assembly was performed by SPAdes Assembler, version 3.14.0 [25] and the assemblies were examined using Quast version 4.0 [26]. Genomes were then annotated with Prokka 1.14.5 [27].

### 2.7. Bacteriophage Genome Annotation

Protein sequences of open reading frames (ORFs), determined with Prokka 1.14.5 [27], were blasted (BLASTp, NCBI, 2019) against non-redundant protein sequences (nr) database. Conserved protein domains of ORF were predicted with Conserved Domain Search (CDD, NCBI) and Pfam [28]. Additionally, remote homologues were also detected using PHYRE2–Protein Homology/analogY Recognition Engine V 2.0 [29]. Presence of signal peptides was analyzed with SignalP-5.0 Server [30]. Remote homologs of phage head-neck-tail module proteins were additionally analyzed on the Virfam sever [31]. Predicted DGR regions were analyzed with myDGR, a server for identification and characterization of diversity-generating retroelements [32].

### 2.8. Phage Classification and Phylogenetic Analysis

Phage life style and classification were computationally analyzed using PHACTS program (https://edwards.sdsu.edu/PHACTS/ accessed on 20 April 2020) [33]. vConTACT2 [34] was used for taxonomic classification using the ViralRefSeq-prokaryotes-v94 database. To determine phage DNA packaging and replication strategy, a phylogenetic analysis of amino acid sequences of TerL–terminase large subunit was made. Sequences of *terL* were downloaded from NCBI and Pfam databases and aligned using the ClustalW [35] program. A phylogenetic tree was then generated with the SeaView Version 5.0.2 [36] integrated phyML using the maximum likelihood approach and GTR nucleotide substitution model. The resulting dendrogram was then visualized with FigTree v1.4.4 (http://tree.bio.ed.ac.uk/software/figtree/ accessed on 20 April 2020).

### 2.9. Identification of Shared Homologous Proteins and Prophage Regions

Based on the closest BLASTp hits of determined ORFs, closest relatives were manually predicted and their bacterial host genomes were examined for prophage presence. Ranges of prophage regions were determined based on the G + C content, predicted functional annotations of neighboring genes, presence of integrase, and other phage specific genes or the identification of repeat sites (attL and attR). Sequences of predicted prophage regions were extracted from host genomes using Artemis software version 1.8 [37], annotated with Prokka 1.14.5 [27] and compared using Easyfig [38]. Protein sequences of ORFs of identified prophages were analyzed for conserved protein domains as described above. Gene synteny in different phage functional gene groups was analyzed.

### 2.10. Single Nucleotide Polymorphism (SNP) Analysis of Potential Phage Target Genes

Reads of original phage host (*B. uniformis* MB18-80) and two derivative strains isolated in lysogeny experiment (MB18-80-K and MB18-80-PH) were mapped to original MB18-80 assembly using BBTools [39]. Sorted BAM files were used for calling SNPs sites using the SAMtools verison 0.1.19 [40]. Mapped reads and SNP sites were also analyzed relative to MB18-80 genome using Artemis software version 1.8 [37].

### 2.11. Tandem Repeats Analysis with Direct Sequencing

Tandem repeats were located and analyzed with Tandem Repeats Finder [41]. Primers (primer F2, 5′-CCTCGGTAATGCTTTCTACG-3′; primer R2, 5′-AGGTAGCCGTAAATGTATCG-3′) were constructed using SnapGene software (GSL Biotech LLC, 2004, Chicago, IL, USA) and were used in a direct Sanger sequencing reaction (40 cycles; using a gDNA as a template and BigDye Terminator v3.1 Cycle Sequencing Kit) to examine if the repeats represent phage genome termini of linear dsDNA phage. Sequencing was performed on 3500 Series Genetic Analyzer (ThermoFisher Scientific, Waltham, MA, USA) and analyzed with Artemis software version 1.8 [37].

### 2.12. Identification of the Isolated Bacteriophages in Metagenomes

Paired-end sequencing reads in fastq format of metagenomics studies under the BioProject accession numbers PRJNA491626, PRJNA268964 and PRJNA278393 were downloaded from The European Nucleotide Archive (ENA) (https://www.ebi.ac.uk/ena, accessed on 26 March 2020). Adaptor removal and quality trimming was conducted by Trimmomatic Version 0.39 [23]. Processed metagenomics reads were mapped to genome assembly of isolated phage using BBTools [39].

### 2.13. Data Availability

The assembled genomes were submitted to the NCBI (https://www.ncbi.nlm.nih.gov, accessed on 26 March 2020) under the Bioproject accession numbers PRJNA636979 (bacterial genomes) and PRJNA638235 (phage genomes).

## 3. Results

### 3.1. Isolation and Phenotypic Characterization of Phages Specific for Bacteroides uniformis

**Figure 1:**
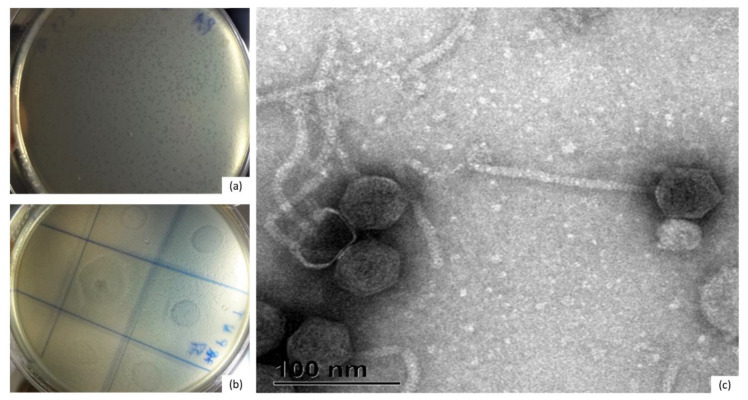
Bacuni phages exhibit *Siphoviridae* morphology. (**a**) Plaque morphology of Bacuni phage F1 formed on *B. uniformis* MB18-33 host lawn after 24 h incubation in sABB agar overlay. (**b**) Lysis-like zones formed on sABB agar overlay after 24 h incubation with host strain *B. vulgatus* MB18-32 in double layer agar overlay (spot assay with enrichment sample). (**c**) Photograph of Bacuni virion obtained by transmission electronic microcopy (scale bar is 100 nm).

In 8 out of 12 *Bacteroides* strains belonging to four species, lysis-like zones were observed. Subsequently, plaques were successfully propagated from two *B. uniformis* strains (Table S1). Circular plaques were formed with diameters ranging from 0.1 to 3 mm (Figure 1a). Four seemingly different bacteriophages were isolated (F1–F4). Phages were stable if stored at 4 °C or −80 °C at high concentration (10^11^ pfu/mL). Subsequent analysis showed that phages F3 and F4 were genetically identical and thus for further experiments only phages F1, F2 and F4 were used.

Host range was tested on all *Bacteroides* strains included in this study (Table S1). In addition to the initially identified *B. uniformis* host strains, lysis-like forms (Figure 1b) were observed with additional representatives of *B. vulgatus, B. uniformis, and B. ovatus,* although we were not able to further propagate the phages.

Attempts to isolate lysogenic *Bacteroides* strains from the formed plaques were not successful. Only 10 out of 35 plates carefully inoculated with the material picked form plaque centers resulted in bacterial growth. These strains were further tested for susceptibility to infection with obtained phages. The experiment was performed three times and no lysogens were detected (discussed in detail below).

Transmission electron microscopy (TEM) analysis showed morphology typical of the *Siphoviridae* family of the *Caudovirales* with icosahedral heads of about 50 nm in diameter and an approximate tail size of 150 × 8 nm (Figure 1c).

### 3.2. Novel B. uniformis Phages Show High Degree of Similarity to Each Other and Belong to a New Genus

The assembled genome lengths of phages F1, F2 and F4 were from 40,421 to 40,653 bp (Table 1). G + C content of phage genome was 51.8% (F1), which is considerably higher than its host genome G + C content (46.3%) obtained from WGS analysis, which is also consistent with *B. uniformis* reference stain G + C content.

The genomes of all four isolated phages were very similar (99.83%) (Table 1). Genomes of phage F1 and F4 differ only in 24 SNPs, of which 18 are condensed in the variable repeat region of DGR, but the phages infect different hosts. Phage F2 shares the same host with phage F1 but deviates from F1 in a 57 bp insertion in putative reverse transcriptase gene of the DGR and in 16 SNPs in the secondary variable repeat region of the DGR.

The isolated phages could not be assigned to any of the known prokaryotic viral clusters using a gene sharing network approach vConTACT2 [34], implying that so far no similar bacteriophages have been reported (Table S2 and Figure S2). Based on no resemblance with known phage genera described to date, phages F1, F2 and F4 were proposed as a new genus, and for the purpose of this paper provisionally named Bacuni—*“BACteroides UNIformis*” phage.

TEM-based classification of Bacuni phages into the *Siphoviridae* family was additionally confirmed in silico using the Virfam server [31], which identifies proteins of the phage head-neck-tail module and assigns phages to the most closely related cluster of phages within the ACLAME [42] database (Figure S3).

### 3.3. Genome Organization of Novel B. uniformis Phages

**Figure 2:**
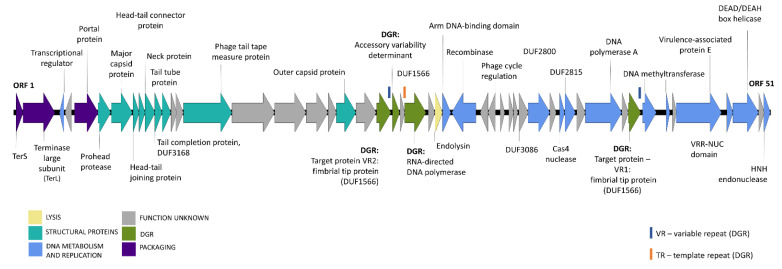
Linear genome map of Bacuni phage F1. Colors of open reading frames correspond to the general predicted functions (see color legend for details). Genes with no functional annotations (hypothetical proteins) are not labeled. Locations of template sequence (TR) and variable repeats (VR) of diversity-generating retroelement (DGR) are marked with orange and red rectangles above associated proteins. The position of ORF 1 was chosen arbitrarily and is not meant to imply the position of natural phage genome ends.

Using automated annotation, 51 open reading frames (ORFs) were predicted in Bacuni genomes. In F4, a small orf was truncated at the end of the assembled sequence, thus 50 orfs were annotated there. Further functional annotation led to the prediction of the potential functions of 34 genes, which could be divided in five common phage functional groups (Figure 2 and Table S3).

Tandem nucleotide repeats were identified in Bacuni phage ORF coding for putative phage tail tape measure protein and direct sequencing was conducted to examine whether repeats in phage genome are terminal, which was not the case. Phylogenetic analysis of large terminase subunit genes (TerL) (Figure S4) indicated that Bacuni phages use rolling circle-concatemer genome replication due to clustering into the group of phages with cohesive ends and 3′-single-strand extensions.

Nine putative structural proteins were identified, including the major capsid protein, prohead protease, a large phage tail tape measure protein with tandem repeats typical for these proteins [43] and four transmembrane helices. The large and small subunits of the terminase and portal protein, which together form a packaging function group, were found located in the close proximity of the structural genes. Bacterial cell wall hydrolytic enzyme, a predicted acetylmuramoyl-L-alanine amidase, was identified as a putative lysin.

Based on a conserved domain search, twelve identified phage genes encode proteins putatively involved in DNA metabolism and replication. Additionally, two genes code for proteins containing domains of unknown function (DUF2800 and DUF2815) that were by bioinformatic approach recently assigned new putative roles in the regulation of phage DNA metabolism [44].

Finally, four functionally annotated genes belong to the diversity-generating retroelement (DGR).

### 3.4. DGR Variability and Host Tropism

Diversity-generating retroelements are recently described genetic elements that use reverse transcription from a donor template repeat (TR) to a recipient variable repeat (VR) in defined target gene. This generates vast numbers of sequence variants (substitutions) in specific target genes [11].

VR sequences of Bacuni phages are located on genes whose products exhibit DUF1566 and/or Fib_succ_major motifs. The Legionella DGR exemplifies the closest studied DGR [45,46]. DGRs found in Bacuni phages belong to a DGR group operating on targets exhibiting a C-type lectin fold [45]. This classification and the presence of DGR elements in Bacuni phages were also confirmed with MyDGR, a server for identification and characterization of diversity-generating retroelements [32].

**Figure 3:**
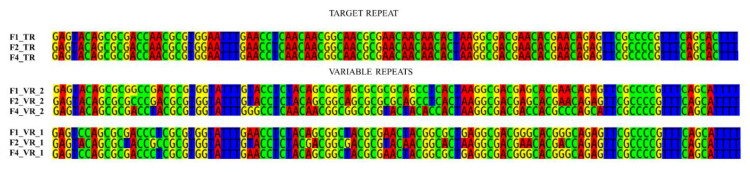
Alignment of the TRs and VRs from isolated Bacuni phages. VR2, located in the close proximity of reverse transcriptase gene, possesses three quarters of all SNP sites differentiating bacteriophages F1 and F4, which infect different hosts.

Bacuni phages have two target genes putatively diversified by DGR. The first target gene (with detected VR1) is located on a distant part of the phage F1 genome (ORF 43) while the second target gene with VR2 (ORF 22) is found in the immediate neighborhood of the core DGR components including reverse transcriptase (RT) (ORF 25), Avd-accessory protein (ORF 23) and the TR containing gene (ORF 24). The variable repeat gene region which is diversified lies at the 3′ end of target genes and codes for the last 35 amino acids. Almost all genetic differences between Bacuni phages are located there (Table 1, Figure 3), leading to 13 amino acid differences in this region between Bacuni phages F1 and F4. The target genes presumably code for motifs involved in host adhesion. Namely, their protein products are confident Phyre2 matches with 60% or higher coverage of the crystal structure of a fimbrial tip protein (bacova_04982) from *B. ovatus* ATCC 8483 [47,48] that was also identified as a DGR target in metagenomes of human stool samples [47].

The observed TR-VR substitutions can be seen in Figure 3 and are, as expected, mutations in adenines. They are most probably the results of induced substitutions mediated by RT.

Despite high genetic similarity, isolated Bacuni phages exhibit different host ranges (Table 1). Since most genetic differences were concentrated in VR regions of DGR target genes located in putative fimbrial tip proteins, we propose that DGRs may influence the host range of Bacuni phages by introducing sequence variations in VR2.

### 3.5. Bacuniphage Similarities to Other Phages and Prophages of Various Anaerobic Bacteria

**Figure 4:**
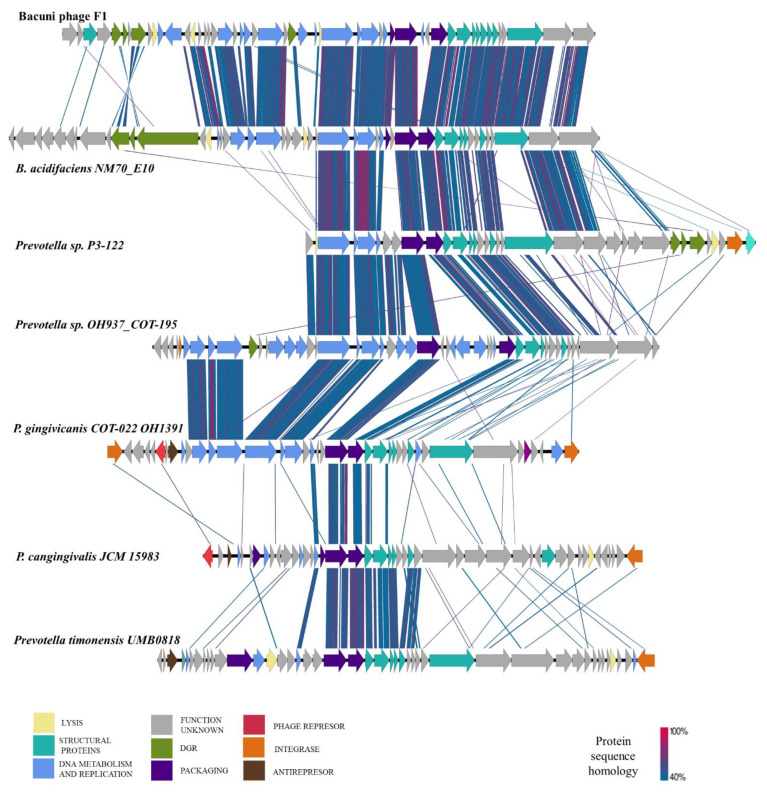
Comparison of genome organization and genomic synteny of Bacuni phages to putative prophage genomes in various bacterial hosts from *Bacteroidales*. BLASTp sequence homology (40% similarity and higer) between Bacuni phage Figure 1. and related prophage regions identified in genomes of *B. acidifaciens, Prevotella sp., P. gingivicanis* and *P. cangingivalis* (see Table 2 for more information) is indicated with a color link. Colors of putative proteins correspond to the general predicted functions (see color legend).

Initially, searches against the NCBI non-redundant database and the Reference Viral Database [49] showed no similarities of Bacuni phages to any known phages at the nucleotide level. BLASTp search, however, revealed some homology to prophage-related gene products encoded in the genomes within the order *Bacteroidales.* Six putative prophage regions were thus identified in assembled bacterial genomes with reliable homologies (Table 2; Figure 4).

Some of the identified prophage regions were found on contig borders and some assemblies were highly fractioned, thus some parts of prophage genomes could have been left out. The putative functions of Prokka annotated prophage ORFs can be seen in Data sheet S1. The identified putative prophage regions have not been described before.

The highest homology (up to 85% amino acid similarity) to proteins of Bacuni phages was observed in putative prophage regions of *B. acidifaciens* NM70_E10 and *Prevotella* sp. P3-122 (Figure 4). They share significant protein homology between two thirds of annotated proteins of various functional clusters including the DGR region and its target region. However, no homologies were found in its putative lysin and recombinase genes.

Protein level homologies found in the remaining identified putative prophage regions of *Prevotella* sp. OH937_COT-195, *Porphyromonas gingivicanis* COT-022 OH1391, *P. cangingivalis* JCM 15,983 and *Prevotella timonensis* UMB0818 were mostly present in structural and packaging functional gene groups (Figure 4).

The prevalence of predicted prophage regions identified in initial screening (Table 2) was further examined in the Genebank nr-database. Minor nucleotide level similarities to the predicted prophage regions were found, with a few exceptions in newly published genomes: nucleotide homology (92%) on 30% of putative *B. acidifaciens* NM70_E10 prophage region length was found in genomes of *B. ovatus* 3725 D1 (CP041395.1), *Bacteroides xylanisolvens* H207 (CP041230.1), and in unidentified phage clone 1013 (JQ680349.1). The whole sequence of predicted *P. cangingivalis* JCM 15,983 prophage was also found in the genome of *P. cangingivalis* ATCC 700,135 isolated in Finland and in *P. cangingivalis* NCTC12856 collected in 1986 and isolated from a fecal sample of *Homo sapiens*.

### 3.6. Identification of Bacuni Phages in Human Gut Virome Database and in Associated Metagenomes

Genome of Bacuni phage F1 was blasted (BLASTn) against the human gut virome database (GVD), a novel database composed of 13,203 unique viral populations obtained from gut metagenomes of 572 individuals from different geographical locations [50]. Matches (roughly 80% nucleotide similarity over more than 80% of the Bacuni phages) were found in contigs originating from two studies [51,52]. Data was further tracked to authentic metagenomic data sets that include metagenomes from Western urban societies and traditional communities [51,52]. Search for reads mapping to the Bacuni phage genome revealed that Bacuni phages were underrepresented in Western data sets analyzed but present in data sets of fecal viromes of Cameroonians with gastroenteritis (Data sheet S2). Up to 6066 reads from the metavirome of a Cameroonian [52] were found to align to Bacuni phage, majority originating from the Kumba region (Data sheet S2). Further analysis showed that those reads cover 31 of the 40 kb Bacuni phage F1 genome confirming earlier BLAST result.

### 3.7. Changes of Host Susceptibility Pattern after Exposure to Bacuni Phage

Assay for detection of lysogenic bacteriophage in *B. uniformis* host strains was conducted (Table S1, Figure S1). Three attempts to isolate potential lysogenic host strains from the formed plaques resulted each in roughly 10 viable derivatives of *B. uniformis* MB18-80 and *B. uniformis* MB18-33. These derivative strains were retested with all three Bacuni phages. Spot assay showed mixed results. Some derivatives were indeed not lysed by any of the phages (representing possible lysogens), while some were resistant to challenging phages but lysed by phages that initially did not lyse the original strain.

Two derivatives of *B. uniformis* MB18-80, host of phage F4, were further selected for WGS: MB18-80-K, a potential lysogen, that was resistant to infection with all tested phages, and second derivative MB18-80-PH that became susceptible to infection with phages F1 and F2, but resistant to F4 (Table S1, Figure S1).

Genome analysis of *B. uniformis* MB18-80-K and *B. uniformis* MB18-80-PH disproved assumptions of lysogenic lifestyle since no parts of Bacuni phage genome were detected in genome of sequenced derivative strains. Comparison of the obtained *B. uniformis* derivative genomes to original host strain indicated SNPs in several biologically relevant genes (Table 3 and Table S4). Genome of immune derivative *B. uniformis* MB18-80-K exhibits SNPs in genes coding for putative restriction enzymes involved in its defense mechanism against invading viruses and in outer membrane transporter complexes most likely involved in the import of large degradation products of proteins or carbohydrates (Table 3 and Table S4). *B. uniformis* MB18-80-PH, in which phage tropism switching was observed, exhibited SNPs in partially overlapping set of genes coding for restriction enzymes, putative porins, peptidoglycan binding proteins, and putative peptidoglycan hydrolase (Table 3 and Table S4).

## 4. Discussion

*Bacteroides* strains are prominent in the human gut microbiome and are known as dietary fiber fermenters that produce short chain fatty acids important for host health [4,53]. As such they are commonly found in globally conserved core gut microbiota [54,55,56,57].

In this study, we describe the isolation and characterization of human gut associated phages infecting *B. uniformis*. As they were essentially not similar to any of the hitherto described phages based on their encoded proteins, we were not able to classify them using VconTACT2. Thus, they may be the first isolated representatives of a new phage genus, provisionally named here a “Bacuni phage”.

The phages, which were isolated from *Bacteroides* enrichment cultures, infected distinct *B. uniformis* strains and possessed complete DGRs. DGRs are composed of a template-dependent reverse transcriptase and accessory proteins that produce mutations in target genes with variable repeats. This introduces variability in the target proteins [45]. The DGR mechanism was first described in *Bordetella* phage BPP-1, in which mutations target phage tail fiber gene to enable bacterial host species switching [58,59]. Phage-encoded DGRs were also found in genomes of isolated temperate phages of intestinal *B. dorei* [12] and *F. prausnitzii* [11]. Moreover, DGRs were detected in defined prophage regions of bacteria belonging to *Bacteroidetes*, *Proteobacteria* and *Firmicutes,* obtained from human-gut associated metagenomes and bacterial genomes [12].

The tropism of Bacuni phages appears to be dependent on interplay of DGR mediated sequence variations of putative phage fimbrial tip proteins and mutations in host genes coding for outer-membrane proteins. Different host ranges between genetically very similar Bacuni phages can be explained with SNP sites condensed in variable repeats of the DGR region, located in a putative fimbrial tip protein, a gene presumably involved in cell adhesion and possibly acting as a cell receptor in Bacuni phages. Both variable repeats lie at the 3′ end of target genes and encompass a region coding for 35 amino acids. Bacuni phages F1 and F4 differ in 13 amino acids in regions coded by the variable repeat 2 within the putative fimbrial tip protein and infect different hosts, while Bacuni phages F1 and F2, that infect the same host, differ in this region only in one amino acid. Given that there are only 6 additional SNPs observed between F1 and F4 outside of the DGR and two of them are synonymous, one can hypothesize that variable repeat 2, located in close proximity of reverse transcriptase, presumably plays a role in Bacuni phage tropism in our experimental setting. These findings correlate with a study where a metagenomic data set from the human microbiome project (HMP) was screened for DGRs [48]. There, the identified variable regions were also localized in a DUF1566 domain coding genes and the target protein showed high protein homology to a pilin tip from *Bacteroides ovatus* [12,47]. The functional consequences of the observed 19 amino acid insertion in the reverse transcriptase of the bacteriophage F2, though probable, are elusive due to the lack of structure information on related proteins.

It appears that DGR contributes to increased adaptability of phages in such complex communities as the human gut, where multiple species of the same genus and several strains of the same species may coexist. This evolutionary advantage may (indirectly) affect microbial diversity and influence health of the associated mammalian host.

To the best of our knowledge, Bacuni phages are the first easily propagated DGR-containing *Bacteroides* bacteriophages [12]. Given their family affiliation, genome size, protein homologies to six putative prophages (Figure 4, Table 2) and their paucity in virome studies, one would presume that Bacuni phages should be temperate. Yet, we were unable to demonstrate lysogenisation in laboratory and the bioinformatic pipeline PHACTS produced inconclusive results.

Viral databases do not contain many genomes of phages infecting dominant gut bacteria and we were initially not able to locate a metagenome/virome that contained sequence reads mapping to Bacuni phages. However, a recently published GVD database improves viral detection rates over NCBI viral RefSeq by nearly 60-fold [50]. An almost-complete Bacuni phage genome was found in GVD originating from intestinal viromes of Cameroonians [52]. Weak signal of reads in metagenomes of traditional Peruvian communities and urban Italian gut metagenomes [51] may indicate that these phages are present at various geographic locations but not abundant enough to be detected with common metagenomics sequencing technologies that are generally not yet optimized to detect bacterial viruses.

Our study sheds light on the feasibility of isolation of phages infecting abundant gut bacteria. If Bacuni phages are proven to be lytic, they could be suitable for use in phage therapy [60,61,62,63]. In vivo studies in rats using commercial phage cocktails showed that phages triggered a cascade reaction that influenced bacterial diversity and composition of gut microbiota [64]. Additional further research may provide phages targeting less-beneficial bacteria in the intestine with a potential therapeutic role on human gut microbiota.

## Figures and Tables

**Table 1:** Comparison of general characteristics of isolated phages belonging to a newly defined genus Bacuni.

Phage	F1	F2	F3 and F4
Bacterial host	*Bacteroides uniformis* MB18-33	*Bacteroides uniformis* MB18-33	*Bacteroides uniformis* MB18-80
No. of predicted ORFs	51	51	50
Assembled genome length (bp)	40,421	40,653	40,112
G + C content (%)	51.8	51.7	51.7
Genetic differences (compared to F1)	Reference	16 SNPs in the DGR, of these 15 in the secondary VR and 57 bp insertion in RT gene	24 SNPs, 18 in DGR primary VR RT identical to RT F1

(RT—reverse transcriptase, VR—variable repeat, SNP—single nucleotide polymorphism, ORF—open reading frame; DGR: Diversity-generating retroelements).

**Table 2:** Comparison of selected genome characteristics between Bacuni phages and putative partially homologues prophage genomes.

Host Strain	Source	Collection Date and Location	Region Length (bp)	No. of Bacuni Homologous Proteins/No. of ORFs	Coverage * (%) —nt Identity (%)	Genome Location and Biosample Accession
*Bacteroides acidifaciens* NM70_E10	*Mus musculus,* colon and cecum	2016, Toronto, Canada	44,986	28/48	45% —71.06%	Node 8 (64,227,109,212) SAMN10878312
*Prevotella* sp. P3-122	*Sus scrofa domesticus*, feces	2014, Slovenia: pig farm Ihan	34,280	Contig 46: 21/35, Contig 76: 5/15	46% —72.82%	Contig 46 (4,434,078,619) Contig 76 (112,007) SAMN07431220
*Prevotella* sp. OH937_COT-195	*Canis lupus*, dog mouth	2012, Leicestershire, UK	38,640	17/47	28% —71.82%	Scaffold20 (316,337,390) SAMN10478691
*Porphyromonas gingivicanis* COT-022 OH1391	*Canis lupus*, dog mouth	2012, Leicestershire, UK	35,922	11/39	23% —70.63%	Contig 6 (1,637,952,300) SAMN03004338
*Porphyromonas cangingivalis* JCM 15983	n.a.	2014, The University of Tokyo	33,481	17/46	33% —67.78%	Node 1 (310,636,586) SAMD00003336
*Prevotella timonensis* UMB0818	*Homo sapiens*, catheter	2015, USA: Maywood, IL	37,867	16/47	4% —69.19%	Node 1 (5,617,794,272) SAMN07511428

* Genome coverage ~ Percent of nucleotide identity (discontiguous megablast) compared to F1.

**Table 3:** Genetic differences in Bacuni phage F4 host MB18-80 derivatives that are immune to infection with Bacuni phages or indicate a tropism switching pattern.

Putative Functionof *B. uniformis* MB18-80 Protein	NCBI Accession * of Closest BLASTp Hit	SNP in *B. uniformis* MB18-80 K (Immune)	SNP in *B. uniformis* MB18-80 PH (Switched Tropism)
Type I restriction-modification system specificity (S) subunit	WP_117795664.1, WP_118086673.1	+	+
TonB-linked outer membrane protein, SusC receptor	EOS06643.1, WP_080597360.1	+	−
Outer-membrane protein OmpA, DUF5082	WP_034528676.1, WP_034528679.1	−	+
Putative porin–exopolysaccharide biosynthesis protein YbjH	WP_034528957.1, WP_120141442.1, WP_147392574.1, WP_147392573.1	−	+

* BLASTp coverage range from 96% to 100%, identity from 99.5% to 100%. + SNP present; − SNP absent.

## Data Availability

The datasets generated for this study can be found in the NCBI (https://www.ncbi.nlm.nih.gov/, accessed on 20 April 2021) under the Bioproject accession numbers PRJNA636979 (bacterial genomes) and PRJNA638235 (phage genomes). Bacuni phages genomes are available under accession numbers: MT635598.1 (Bacuni phage F1), MT806185.1 (Bacuni phage F4) and MT806186.1 (partial genome; Bacuni phage F2), MT806187.1 (partial genome; Bacuni phage F2).

## Supplementary Materials

The following are available online at https://www.mdpi.com/article/10.3390/microorganisms9050892/s1, Table S1: List of Bacteroides strains, Table S2: vcontact2 results, the phage F1 is named 3P11 and the phage F4 8POS, Table S3: Putative functions of ORFs in prophages, Table S4: Genetic differences in genes of Bacuni host derivatives, Data sheet S1: Putative functions of ORFs in prophages, Data sheet S2: Abundance of reads aligning to Bacuni F1 in gut metagenomes, Figure S1: Schematic overview of lysogenic assay, Figure S2: Vcontact clusters, the phage F1 is named 3P11 and the phage F4 8POS, Figure S3: Virfam server generated identification, Figure S4: Phylogenetic analysis of TerL.
