# Supplementary figures and images for: Novel Siphoviridae Bacteriophages Infecting Bacteroides uniformis Contain Diversity Generating Retroelement
Source: Microorganisms. 2021 Apr 21;9(5):892. doi: 10.3390/microorganisms9050892 (PMC8143477; doi:10.3390/microorganisms9050892)

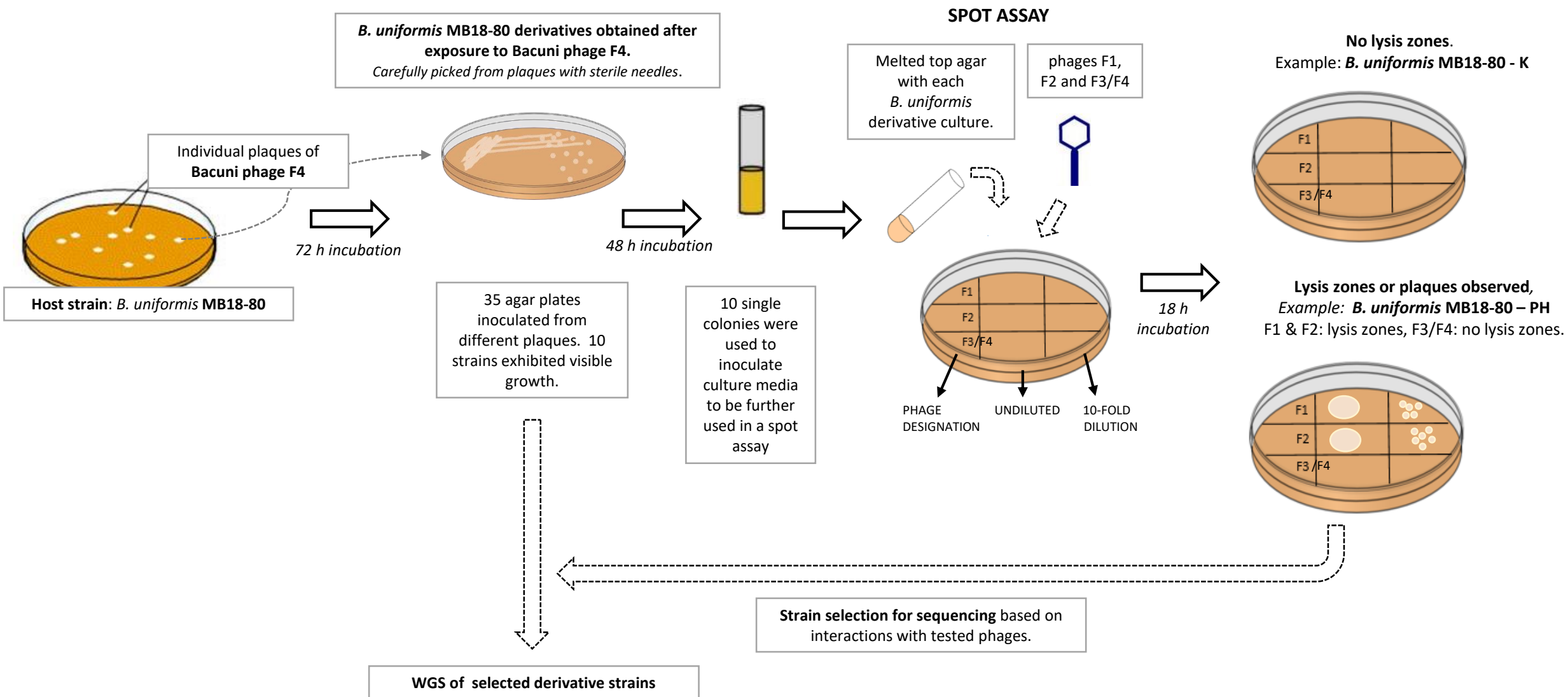

Supplement: Supplementary file 1 [file microorganisms-09-00892-s001.zip › Figure S1_Schematic overview of lysogenic assay.pdf]

## Slide 1
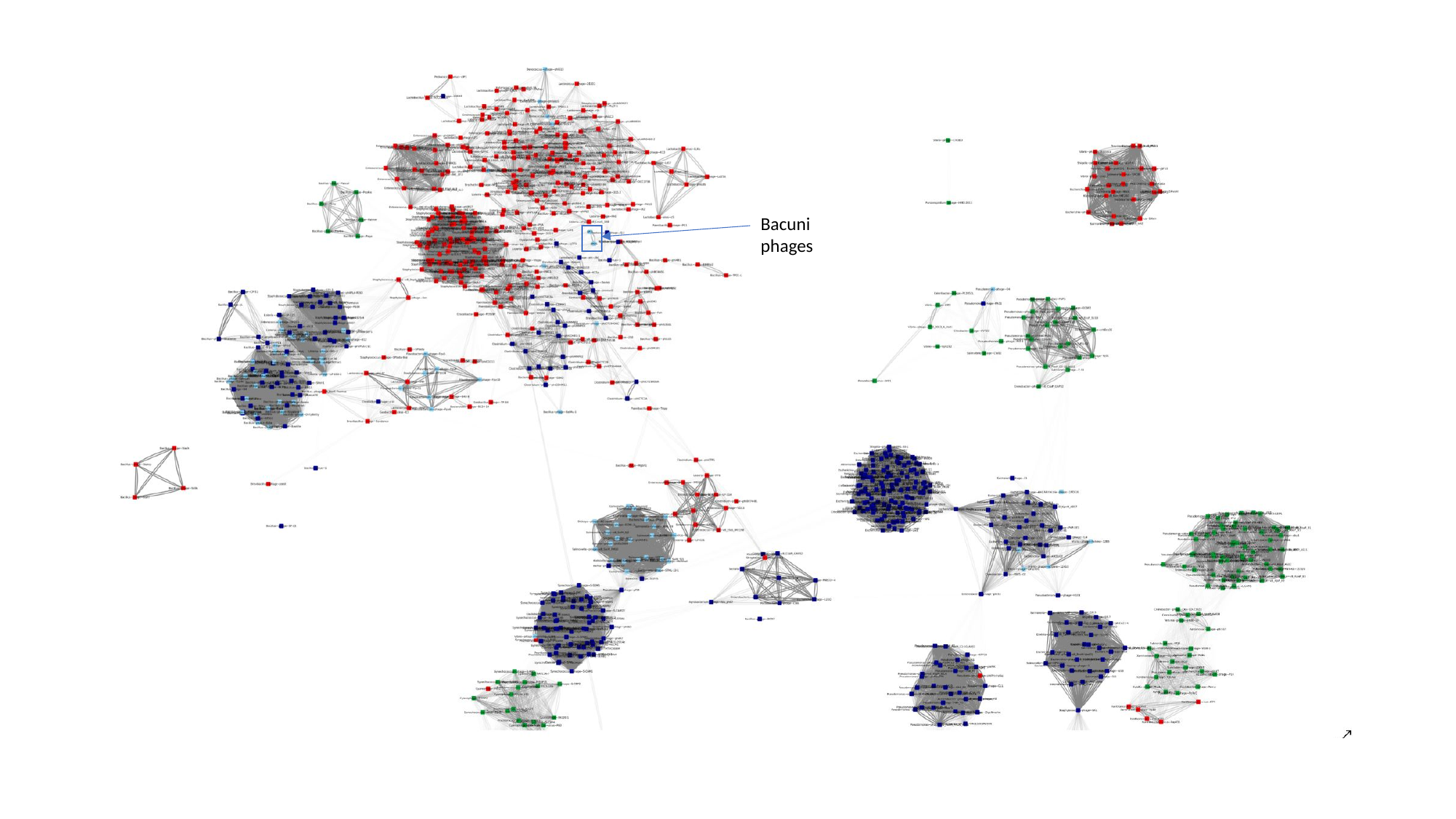

Bacuni phages

Supplement: Supplementary file 1 [file microorganisms-09-00892-s001.zip › Figure S2_Vcontact clusters.pptx]

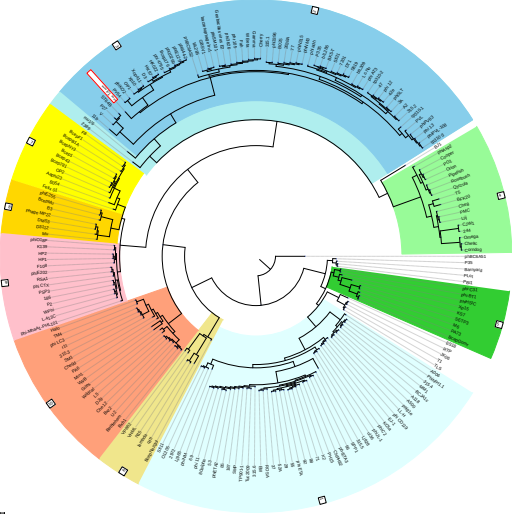

Supplement: Supplementary file 1 [file microorganisms-09-00892-s001.zip › Figure S3_Virfam server generated identification.pdf]

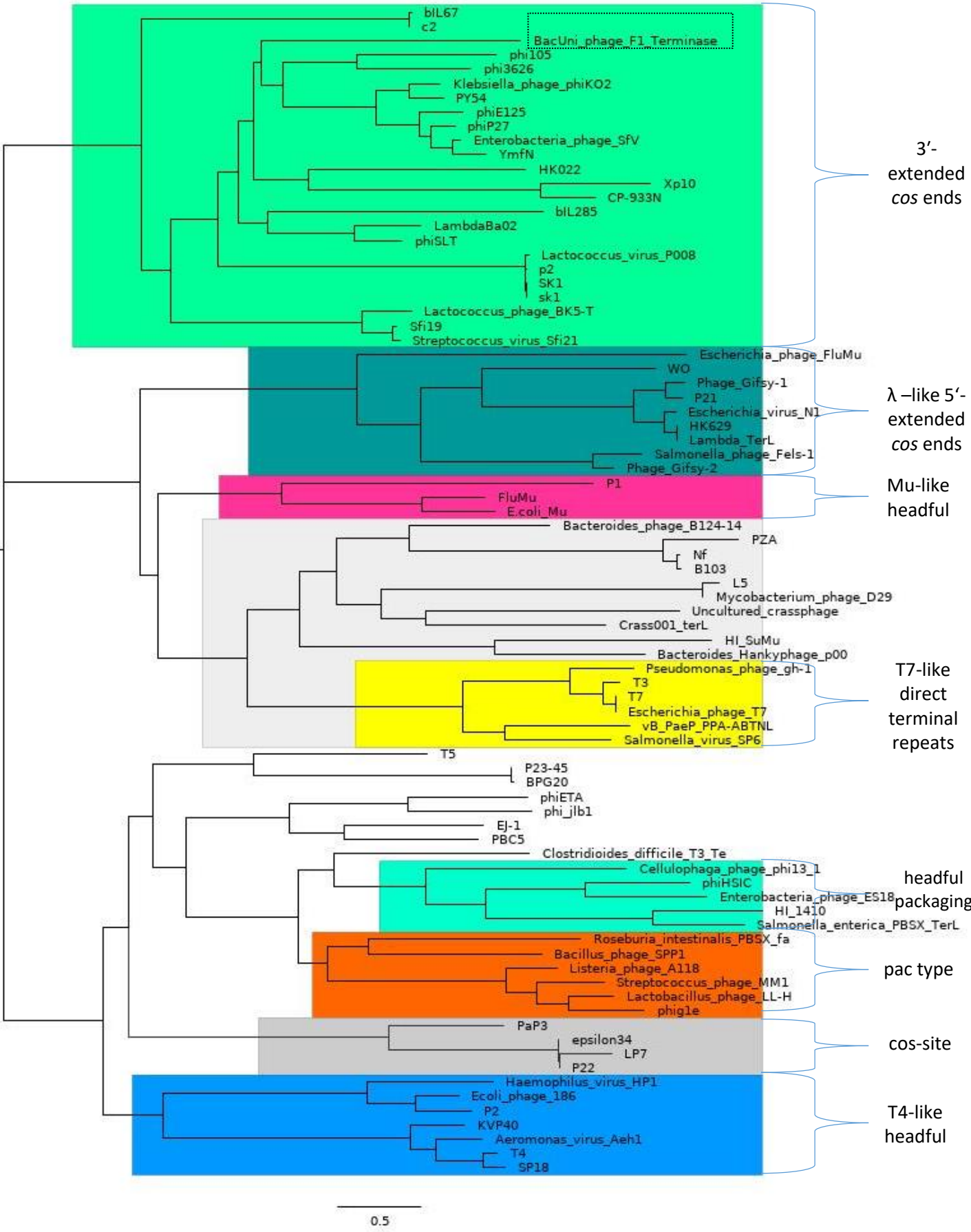

Supplement: Supplementary file 1 [file microorganisms-09-00892-s001.zip › Figure S4_Phylogenetic analysis of TerL.pdf]
